# The Small Molecule Alpha-Synuclein Aggregator, FN075, Enhances Alpha-Synuclein Pathology in Subclinical AAV Rat Models

**DOI:** 10.3390/biom11111685

**Published:** 2021-11-12

**Authors:** Rachel Kelly, Andrew G. Cairns, Jörgen Ådén, Fredrik Almqvist, Alexis-Pierre Bemelmans, Emmanuel Brouillet, Tommy Patton, Declan P. McKernan, Eilís Dowd

**Affiliations:** 1 Pharmacology & Therapeutics and Galway Neuroscience Centre, National University of Ireland Galway, H91 W5P7 Galway, Ireland; r.kelly35@nuigalway.ie (R.K.); t.patton1@nuigalway.ie (T.P.); declan.mckernan@nuigalway.ie (D.P.M.); 2 Department of Chemistry, Umeå University, 901 87 Umeå, Sweden; andrewgcairns@gmail.com (A.G.C.); jorgen.aden@umu.se (J.Å.); fredrik.almqvist@umu.se (F.A.); 3 Université Paris-Saclay, CEA, CNRS, Laboratoire des Maladies Neurodégénératives, MIRCen, F-92265 Fontenay-aux-Roses, France; alexis.bemelmans@cea.fr (A.-P.B.); emmanuel.brouillet@cnrs.fr (E.B.)

**Keywords:** Parkinson’s, alpha-synuclein, adeno-associated virus, AAV, phospho-alpha-synuclein

## Abstract

Animal models of Parkinson’s disease, in which the human α-synuclein transgene is overexpressed in the nigrostriatal pathway using viral vectors, are widely considered to be the most relevant models of the human condition. However, although highly valid, these models have major limitations related to reliability and variability, with many animals exhibiting pronounced α-synuclein expression failing to demonstrate nigrostriatal neurodegeneration or motor dysfunction. Therefore, the aim of this study was to determine if sequential intra-nigral administration of AAV-α-synuclein followed by the small α-synuclein aggregating molecule, FN075, would enhance or precipitate the associated α-synucleinopathy, nigrostriatal pathology and motor dysfunction in subclinical models. Rats were given unilateral intra-nigral injections of AAV-α-synuclein (either wild-type or A53T mutant) followed four weeks later by a unilateral intra-nigral injection of FN075, after which they underwent behavioral testing for lateralized motor functionality until they were sacrificed for immunohistological assessment at 20 weeks after AAV administration. In line with expectations, both of the AAV vectors induced widespread overexpression of human α-synuclein in the substantia nigra and striatum. Sequential administration of FN075 significantly enhanced the α-synuclein pathology with increased density and accumulation of the pathological form of the protein phosphorylated at serine 129 (pS129-α-synuclein). However, despite this enhanced α-synuclein pathology, FN075 did not precipitate nigrostriatal degeneration or motor dysfunction in these subclinical AAV models. In conclusion, FN075 holds significant promise as an approach to enhancing the α-synuclein pathology in viral overexpression models, but further studies are required to determine if alternative administration regimes for this molecule could improve the reliability and variability in these models.

## 1. Introduction

Many of the animal models of Parkinson’s disease that have been developed to date involve the administration of a toxin either systemically or cerebrally, such as the direct toxins 6-hydroxydopamine (6-OHDA) and 1-methyl-4-phenyl-1,2,3,6-tetrahydropyridine (MPTP); the pesticide, rotenone, and the herbicide, paraquat; and inflammagens such as LPS and poly I:C [1]. However, while undoubtedly useful, these toxin-based models are problematic in that they are usually not progressive, and therefore generally not optimal for testing potential therapeutic interventions. Moreover, they do not replicate all the neuropathological changes that are occurring in the disease state, often just inducing nigrostriatal degeneration in a molecular manner that is not necessarily relevant to the disease, and rarely incorporating α-synuclein pathology. Viral vector-mediated overexpression of α-synuclein was first introduced as a potential approach to modelling Parkinson’s disease just a few years after the implication of α-synuclein in the pathogenesis of the disease as the major component of Lewy bodies [2]. Deniz Kirik and colleagues employed adeno-associated viral (AAV) vectors that expressed either wild-type or A53T mutated human α-synuclein and injected them into the substantia nigra of rats [3]. They observed a substantial loss of nigral dopaminergic neurons and consequently a loss of striatal dopamine that was accompanied by motor dysfunction in animals in which the dopamine neuron loss exceeded a critical threshold of 50–60%. They also noted the presence of α-synuclein-positive inclusions and dystrophic neurites similar to those seen in Parkinson’s disease patients. Subsequently, AAV vectors have been used with similar success in mice [4,5] and primates [6,7]. Lentiviral vectors have also been utilized, but to a lesser extent as they produce a lower transgene expression in midbrain dopamine neurons and a relatively reduced death of neuronal cells [8,9]. 

However, despite the advantages of this viral vector-induced α-synuclein overexpression model, it has its limitations: primarily, a very slowly developing pathology and a high degree of variability. Although this variability can partially be attributed to the different capsid serotypes and different titers of vectors used by distinct research groups, this does not fully explain the disparities in neuronal loss [10]. Even within a singular study, discernible inconsistencies have been noted with regard to the extent of observable neurodegeneration between animals [3]. In addition, the levels of α-synuclein expression required to produce dopaminergic degeneration are much higher (up to 4–5 times) than those present in the human disease [11], rendering it physiologically less relevant. These limitations are substantial disadvantages which indicate an improved α-synuclein model of Parkinson’s disease is required. 

FN075 is a novel peptidomimetic small molecule that has been shown to inhibit the production of functional curli or bacterial amyloid fibers, but also to stimulate the aggregation of α-synuclein [12,13] (for structure of the molecule, see [13]). In vivo, a single injection of FN075 into the brains of mice caused impairments in motor function and induced a significant reduction of tyrosine-hydroxylase positive neurons at six months. In α-synuclein knockout mice, there were no differences in neuronal numbers, indicating that this was an α-synuclein-mediated effect [14]. Previous members of our group studied FN075 in the context of the interaction between viruses and α-synuclein, and found that priming with a viral mimetic significantly exacerbated the α-synuclein aggregate-induced behavioral and neuropathological effects that were mediated by FN075. Specifically, sequential exposure to poly I:C and FN075 caused significant nigrostriatal neurodegeneration and microgliosis, and significant impairments in forelimb kinesis and sensorimotor integration [15].

Given the aforementioned problems with the current AAV-α-synuclein model, and the promising results reported from studies germane to the small molecule FN075, we sought to investigate if intra-nigral administration of AAV-α-synuclein followed by FN075 would enhance or precipitate α-synucleinopathy, nigrostriatal pathology and motor dysfunction in AAV-α-synuclein models of Parkinson’s disease.

## 2. Materials and Methods

### 2.1. Animals & Ethical Statement

Eighty adult female Sprague-Dawley rats (Charles River, Margate, UK) were used in this research. All procedures involving the use of animals were in compliance with the European Union Directive 2010/63/EU and the Irish Statutory Instrument S.I. No. 543 of 2012; were approved by the Animal Care and Research Ethics Committee (ACREC) of the National University of Ireland, Galway; and were completed under Project and Individual Authorizations issued by the Irish Health Products Regulatory Authority. Animals were housed in pairs, on a 12:12 light:dark cycle, with 19–23 °C temperature and 40–70% humidity. They were provided with water and food ad libitum throughout the course of the experiments except for 24 h before the Corridor Test was performed in which their food ration was reduced (to ensure they were driven to perform this food-motivated test). All behavioral testing and post mortem analyses were completed by a researcher blinded to the treatment of the rats. 

### 2.2. Experimental Design

Two separate experiments were completed: one using AAV vectors expressing the wild-type human α-synuclein transgene and one using AAV vectors expressing the A53T mutant human α-synuclein transgene (Table 1), which is associated with a familial inherited form of Parkinson’s disease [16]. In both studies, rats first underwent behavioral habituation to the Stepping, Whisker and Corridor Tests before being randomly assigned to receive unilateral intra-nigral injections of AAV-α-synuclein (or AAV-GFP as a control). Four weeks later, the animals received an intra-nigral injection of the α-synuclein aggregating molecule, FN075 (or its vehicle as a control). Animals were tested for motor dysfunction in the Stepping, Whisker and Corridor Tests prior to AAV surgery as well as at 4 weeks and 20 weeks post-AAV surgery. 20 weeks post-AAV surgery the animals were sacrificed by transcardial perfusion-fixation under terminal pentobarbital anesthesia for immunohistochemical analyses.

### 2.3. AAV Virus Production

AAV6 recombinant genomes encoding wild-type and A53T mutant human α-synuclein, or GFP (green fluorescent protein), under the transcriptional control of the PGK1 (phosphoglycerate kinase) promoter were pseudotyped in serotype 6 capsids as previously described [17,18]. In brief, viral particles were produced by co-transfection of HEK-293T cells with an adenovirus helper plasmid (pXX6-80), an AAV packaging plasmid carrying the rep2 and cap6 genes, and a plasmid encoding a recombinant AAV_2_ genome containing the transgene expression cassette. Seventy-two hours after transfection, viral particles were purified and concentrated from cell lysates and supernatants by ultracentrifugation on an iodixanol density step gradient, followed by dialysis against PBSMK buffer (0.5 mM MgCl_2_ and 1.25 mM KCl in PBS). The concentration of vector stocks was estimated by real-time PCR and expressed as vector genomes per μL of concentrated stocks (vg/μL). On the day of surgery, the vectors were diluted in PBS with 0.01% Pluronic F-68 to the appropriate titer.

### 2.4. Surgery

All surgery was performed under aseptic conditions under isoflurane anesthesia (5% in O_2_ for induction, ~2% in O_2_ for maintenance) in a stereotaxic frame with the nose bar set at −2.3 mm. For the AAV surgery, all rats received a dual intra-nigral injection unilaterally in the substantia nigra (at the coordinates AP −4.8 & −5.8, ML +2.0 and DV −7.2). The titer was 1.67 × 10^10^ vg/µL for surgeries involving the wild-type α-synuclein vector and 1.33 × 10^10^ vg/µL for surgeries involving the A53T mutant α-synuclein vector. Infusions were carried out at a rate of 0.5 µL/min with a total volume of 3 µL per site (2 sites per rat), and a further 5 min were then allowed for diffusion. For the FN075 surgery, all rats received an intra-nigral injection unilaterally into the substantia nigra of FN075 (1 mM) or its vehicle (100 μM imidazole in PBS + 0.5% DMSO; at the coordinates AP −5.3, ML +2.0 and DV −7.2). Infusions were carried out at a rate of 1 µL/min with a total volume of 4 µL, and a further 2 min were then allowed for diffusion.

### 2.5. Behavioral Testing

To assess the impact of the sequential intra-nigral infusions on motor function, the rats were assessed for motor impairments using the Stepping Test of forelimb akinesia [19], the Whisker Test of sensorimotor integration [20] and the Corridor Test of sensorimotor neglect [21,22]. In the Stepping Test, rats were held with both hindlimbs and one forelimb restrained and then guided across a table surface at a steady pace (90 cm in ~5 s). The number of adjusting steps made by the free forelimb was counted in both the backhand and forehand directions on the ipsilateral and contralateral sides (for space considerations, forehand and backhand steps were combined and only data from the contralateral side are shown in the results). In the Whisker Test, rats were held with both hindlimbs and one forelimb restrained while their whiskers on the side of the unrestrained limb were brushed against the side of a table 10 times. The number of vibrissae-elicited forelimb placings was recorded on the ipsilateral and contralateral sides (for space considerations, only placings on the contralateral side are shown in the results). In the Corridor Test, rats were placed in a long corridor and allowed to freely retrieve CocoPops^®^ from pots placed at regular intervals on the left and right-hand sides of the corridor. Trials were deemed completed once the animal made a total of 20 retrievals or after 5 min had elapsed. The number of retrievals made from both the ipsilateral and contralateral sides was counted, and contralateral retrievals were expressed as a percentage of total retrievals made. 

### 2.6. Euthanasia and Tissue Processing

Animals were euthanized 20 weeks post-AAV surgery by transcardial perfusion-fixation under terminal pentobarbital anesthesia (50 mg/kg). Brains were post-fixed in 4% paraformaldehyde for 24 h before being cryoprotected in a 30% sucrose solution with 0.1% sodium azide. Serial brain sections (30 µm) were cut using a freezing sledge microtome (Bright, Cambridgeshire, UK) and collected in a series of 12.

### 2.7. Immunohistochemistry

Free-floating immunohistochemistry was performed using the streptavidin-biotin-peroxidase method as previously described [23,24]. In brief, sections were quenched in a solution containing 3% hydrogen peroxide and 10% methanol in distilled water to eliminate endogenous peroxidase activity. Non-specific antibody binding was blocked by incubation in a solution containing 3% normal horse serum or normal goat serum (depending on the host species of the secondary antibody) in tris-buffered saline (TBS) with 0.2% Triton X-100 at room temperature for 1 h. The primary antibody (Mouse anti-human-α-synuclein, 1:10,000, Millipore 36-008; Rabbit anti-phospho-α-synuclein (S129), 1:5000, Abcam ab51253; Rabbit anti-α-synuclein-aggregate-specific, 1:4000 [25], Abcam ab209538; Mouse anti-tyrosine hydroxylase, 1:1000, Millipore MAB318) was diluted in 1% serum in TBS with 0.2% Triton X-100 and allowed to incubate with the sections overnight. Sections were then incubated with the appropriate biotinylated secondary antibody (Horse anti-mouse, 1:200, Vector BA-2001; Goat anti-rabbit, 1:200, Jackson ImmunoResearch 111-065-144) with 1% serum for 3 h. A streptavidin-biotin-horseradish peroxidase solution (Vector PK-4000) was subsequently added to sections and allowed to incubate for 2 h. Development of the staining was performed using a 0.5% diaminobenzidine tetrahydrochloride (DAB) (Sigma D5637) solution in TBS containing 0.3 µL/mL of hydrogen peroxide. Sections were mounted onto gelatin-coated slides, dehydrated in an ascending series of alcohols, cleared in xylene and coverslipped using DPX mountant. 

### 2.8. Image Analysis

Images were taken of immunostained sections using a VS120 Virtual Slide Microscope (Olympus, Southend-on-Sea, UK). All image analyses were performed using ImageJ software (ImageJ v1.52a, National Institute of Health, Bethesda, MD, USA). In each case, three images were selected along the rostrocaudal axis of the striatum or nigra in an unbiased manner based on their distance from bregma (striatal AP coordinates: +0.7, +1.0, +1.2 mm; nigral AP coordinates −5.6, −5.8, −6.04 mm). The optical density of the staining of α-synuclein in the substantia nigra and the striatum, of phosphorylated α-synuclein in the substantia nigra, and of tyrosine hydroxylase in the striatum was measured using ImageJ software. To do so, the mean grey value of both the intact side and the lesioned side was measured. These were then converted to optical densities by applying the conversion formula in ImageJ: optical density = log_10_ (255/mean grey value). Optical densities were then expressed as a percentage of the intact side. For quantification of substantia nigra cell body counts, the number of tyrosine hydroxylase immunopositive cell bodies in the substantia nigra were counted on both the ipsilateral and contralateral sides according to distinct boundaries. Specifically, immune-positive cells in the substantia nigra pars reticulata, pars lateralis and pars compacta were counted and immune-positive cells in the ventral tegmental area (VTA) were excluded. Cell counts data were expressed as a percentage of the intact side. The number of pS129-α-synuclein-positive accumulations in the substantia nigra were also counted on the side of the brain ipsilateral to the lesion surgery. Data were expressed as the average number of pS129-α-synuclein-positive accumulations in the three sections analysed.

### 2.9. Statistical Analysis

Statistical analyses were carried out using GraphPad Prism software (Version 9.1.2.). Prior to analyses, data were tested for normality using Shapiro-Wilk’s test and homogeneity of variance using Brown-Forsythe’s test to determine if the data was parametric or non-parametric. All parametric data were expressed as mean ± standard error of the mean. If data were parametric, one-way ANOVA was used to compare the mean of more than two groups on one factor, with post hoc Tukey analyses used to determine where the difference lay between groups. Behavioral data were measured using a two-way ANOVA with repeated-measures (with within subject-subject factor of time and between subject-factor of group) with post hoc Tukey analyses. Results were deemed significant if *p* < 0.05. Throughout the results, the main outcome from the ANOVA is given in the text while the outcome of any post hoc analysis is shown in the relevant figure and explained in the corresponding legend. 

## 3. Results

### 3.1. AAV-α-Synuclein Administration Induced Significant Alpha-Synuclein Expression

**Figure 1:**
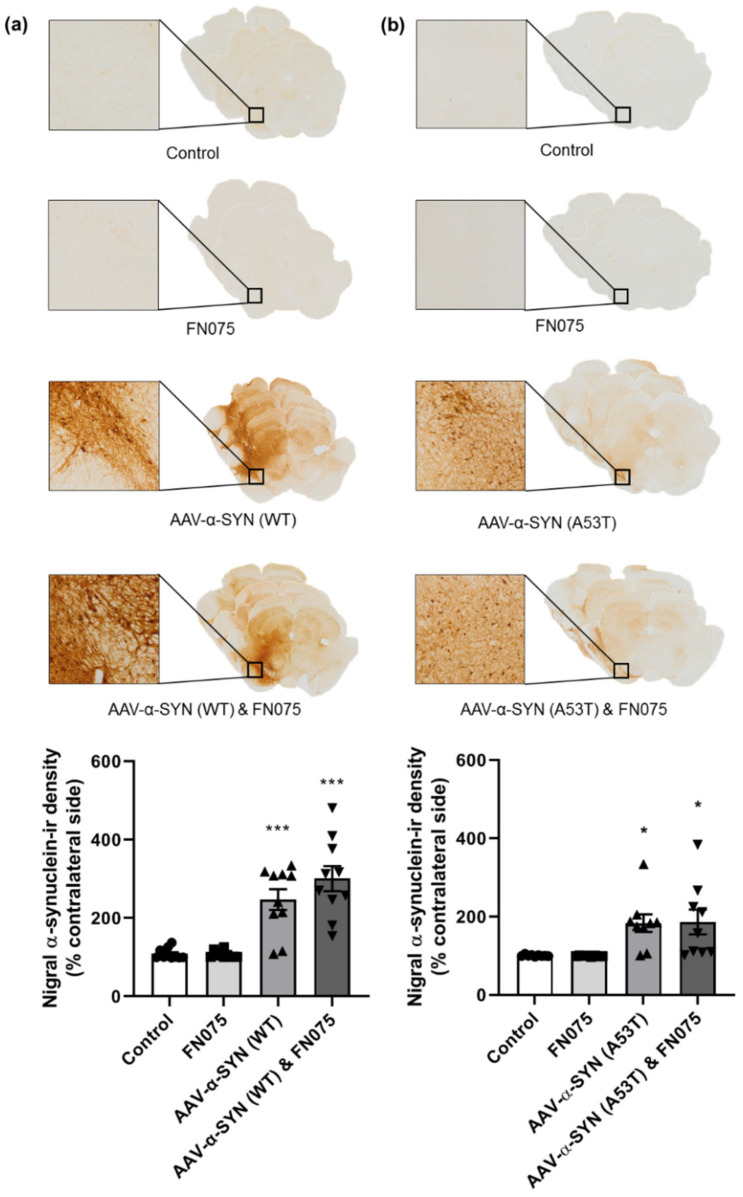
Immunohistological assessment of the impact of the AAV-α-synuclein vectors alone or in combination with FN075 on the expression of human α-synuclein in the substantia nigra. Both (**a**) AAV-α-SYN (WT) and (**b**) AAV-α-SYN (A53T), either alone or in combination with FN075, caused a significant overexpression of human α-synuclein in the substantia nigra. Data are represented as mean ± SEM with *n* = 8–10 animals per group. * *p* < 0.05, *** *p* < 0.001 vs. Control by one-way ANOVA with post hoc Tukey.

We first sought to investigate if administration of the AAV-α-synuclein vectors (AAV-α-SYN) induced the overexpression of human α-synuclein in the nigrostriatal pathway. Immunohistochemical staining for human α-synuclein verified there was widespread α-synuclein expression in the substantia nigra and across the midbrain, in both the case of the AAV-α-SYN (WT) vector (Figure 1a; Group, *F*_(*3*,*36*)_ = 22.11, *p* < 0.05) and the AAV-α-SYN (A53T) vector (Figure 1b; Group, *F*_(*3*,*31*)_ = 5.94, *p* < 0.05). As expected, this effect was restricted to groups that received the AAV-α-synuclein injection, and animals that were administered the AAV-GFP control vector injection showed no α-synuclein expression. The α-synuclein aggregating molecule, FN075, did not affect the density of human α-synuclein immunohistochemical staining, either alone or in combination with either AAV-α-synuclein vector. 

**Figure 2:**
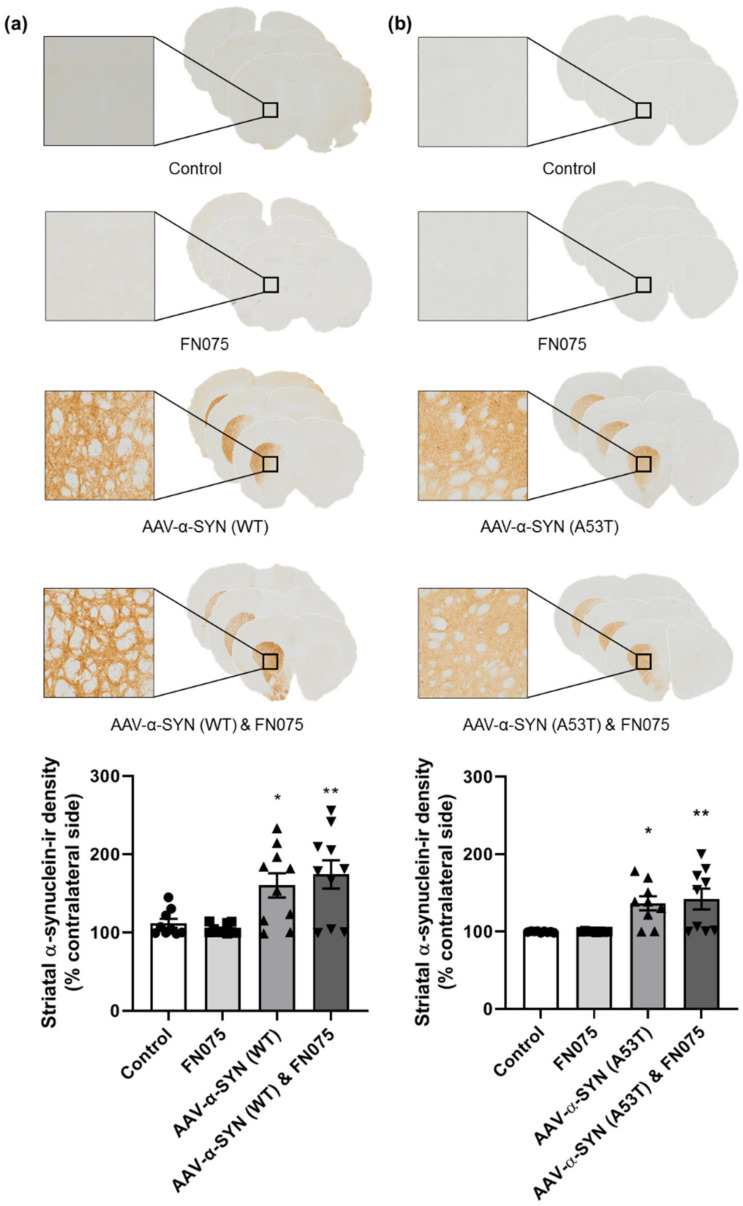
Immunohistological assessment of the impact of the AAV-α-synuclein vectors alone or in combination with FN075 on the expression of human α-synuclein in the striatum. (**a**) AAV-α-SYN (WT) and (**b**) AAV-α-SYN (A53T), either alone or in combination with FN075, caused a significant overexpression of human α-synuclein in the striatum. Data are represented as mean ± SEM with *n* = 8–10 animals per group. * *p* < 0.05, ** *p* < 0.01 vs. Control by one-way ANOVA with post hoc Tukey.

In the striatum, a similar effect was observed. The α-synuclein viral vector induced widespread α-synuclein expression ipsilateral to the side of injection compared to the control group in both the AAV-α-SYN (WT) experiment (Figure 2a; Group, *F*_(*3*,*35*)_ = 7.76, *p* < 0.05) and the AAV-α-SYN (A53T) experiment (Figure 2b; Group, *F*_(*3*,*31*)_ = 7.58, *p* < 0.05), but FN075 did not have any additive effect on the density of staining. 

### 3.2. FN075 Significantly Increased Phosphorylation of Alpha-Synuclein at Serine 129

After verifying that the AAV-α-synuclein vectors caused the overexpression of human α-synuclein in the nigrostriatal pathway, we sought to investigate if the subsequent administration of FN075 affected the form of the α-synuclein protein. α-Synuclein may contribute to Parkinson’s disease pathology in a number of ways, and the presence of a specific form of α-synuclein, phosphorylated at serine 129 (pS129-α-synuclein), has been implicated in the pathogenesis of Parkinson’s disease [26,27,28]. Therefore, we carried out a further immunohistochemical stain for α-synuclein, specific for pS129-α-synuclein.

**Figure 3:**
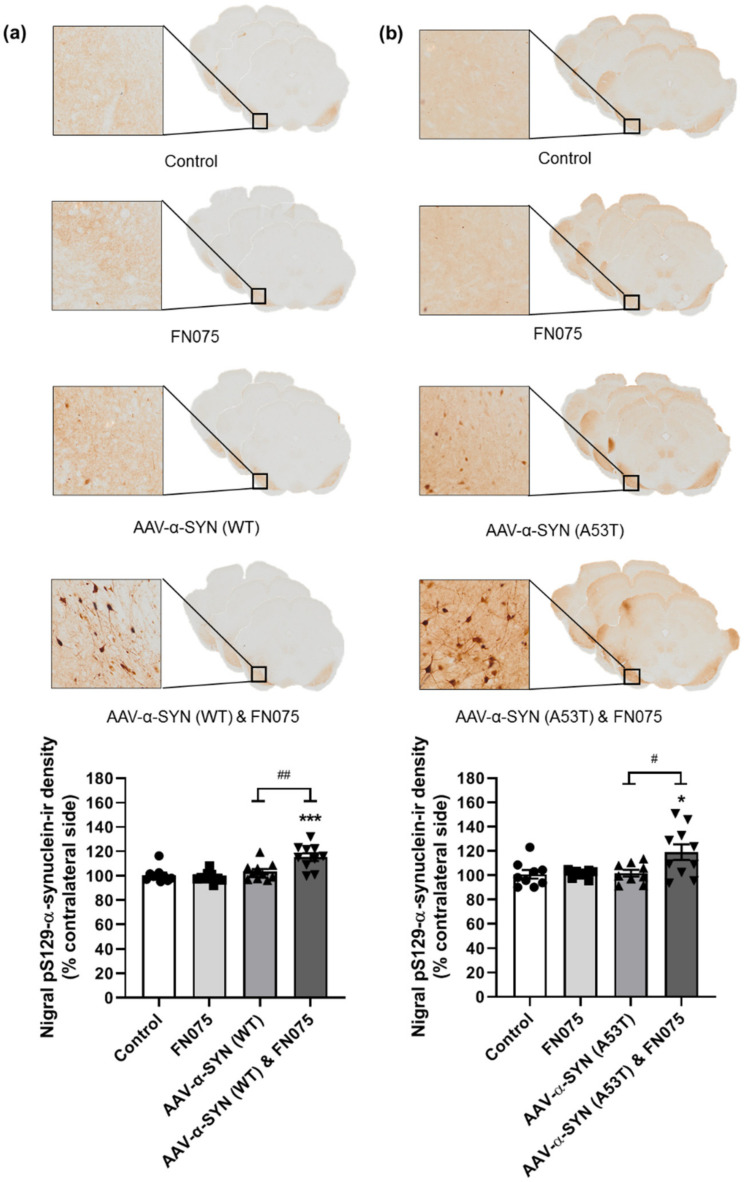
Immunohistological assessment of the impact of FN075 on the phosphorylation of α-synuclein at serine 129 in the substantia nigra. FN075 in combination with (**a**) AAV-α-SYN (WT) or (**b**) AAV-α-SYN (A53T) induced a significant increase in the density of pS129-α-synuclein in the substantia nigra. Data are represented as mean ± SEM with *n* = 8–10 animals per group. * *p* < 0.05, *** *p* < 0.001 vs. Control; # *p* < 0.05, ## *p* < 0.01 vs. corresponding AAV-α-synuclein group by one-way ANOVA with post hoc Tukey.

**Figure 4:**
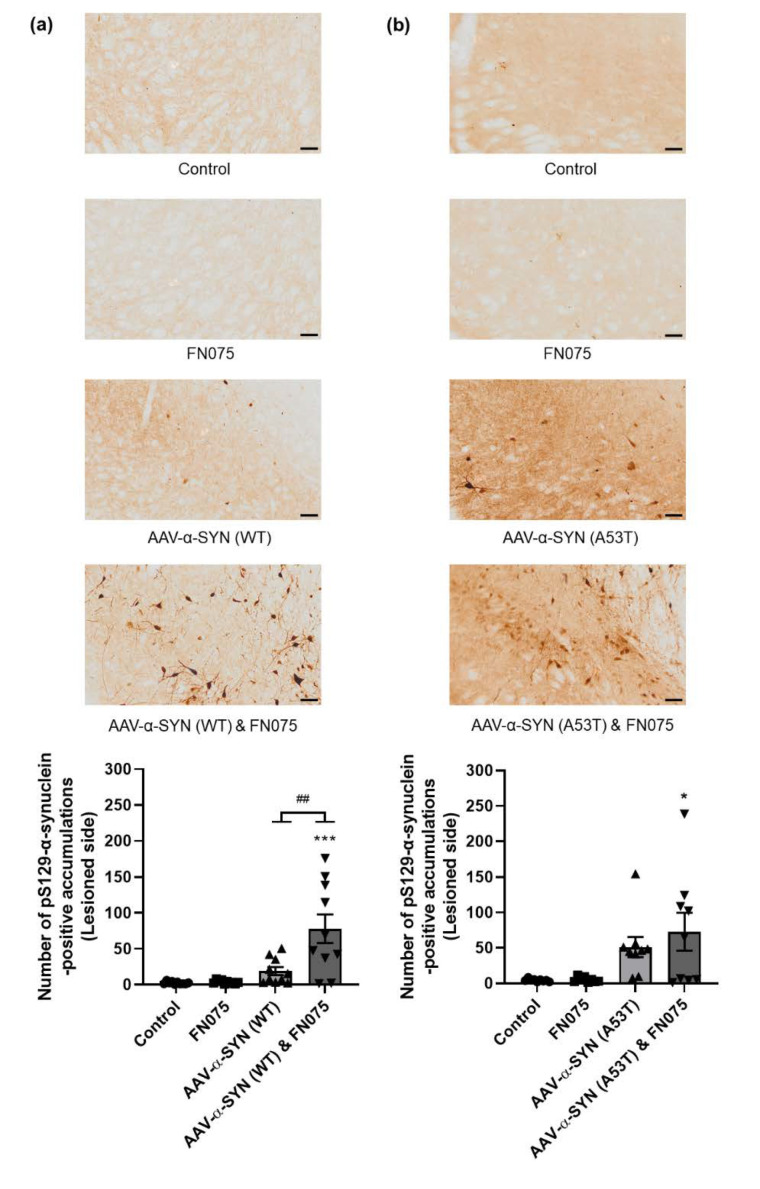
Immunohistological assessment of the impact of FN075 on the accumulation of phosphorylated α-synuclein in the substantia nigra. FN075 in combination with (**a**) AAV-α-SYN (WT) or (**b**) AAV-α-SYN (A53T) induced a significant increase in the number of cells with pS129-α-synuclein accumulations in the substantia nigra. Scale bars represent 50 μm. Data are represented as mean ± SEM with *n* = 8–10 animals per group. * *p* < 0.05, *** *p* < 0.001 vs. Control; ## *p* < 0.01 vs. corresponding AAV-α-synuclein group by one-way ANOVA with *post hoc* Tukey.

Immunostaining revealed a significant increase in the density of pS129-α-synuclein only in the group where AAV-α-synuclein and FN075 were sequentially administered. This was true for both the AAV-α-SYN (WT) study (Figure 3a; Group, *F*_(*3*,*35*)_ = 10.70, *p* < 0.05) and the AAV-α-SYN (A53T) study (Figure 3b; Group, *F*_(*3*,*32*)_ = 4.74, *p* < 0.05). In addition to assessing the density of staining in the substantia nigra, we also counted the number of nigral cell bodies in which pathological pS129-α-synuclein had accumulated. This revealed a build-up of cellular pS129-α-synuclein only in the group with sequential administration of AAV-α-synuclein and FN075 whether the α-synuclein protein was wild-type (Figure 4a; Group, *F*_(*3*,*36*)_ = 11.92, *p* < 0.05) or mutant (Figure 4b; Group, *F*_(*3*,*31*)_ = 4.85, *p* < 0.05).

**Figure 5:**
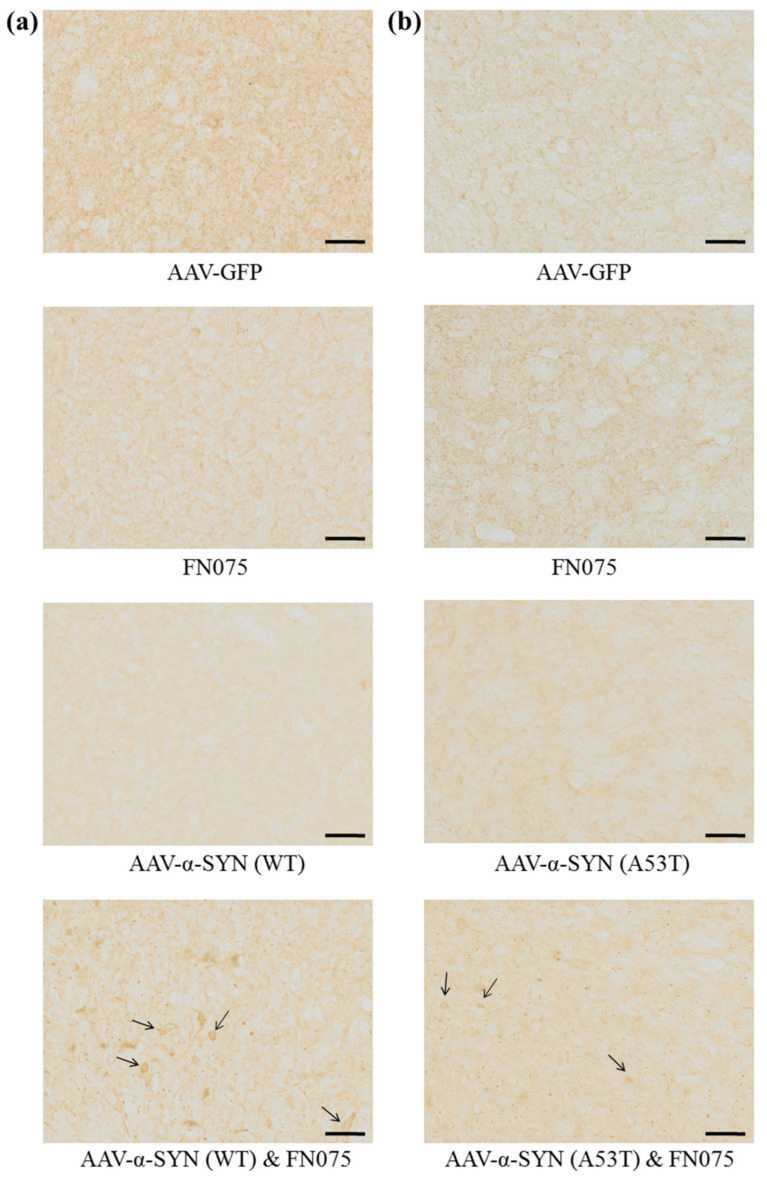
Immunohistological visualization of the impact of the small molecule FN075 on the expression of α-synuclein aggregates in the substantia nigra. FN075 in combination with (**a**) AAV-α-SYN (WT) or (**b**) AAV-α-SYN (A53T) induced the aggregation of α-synuclein in the substantia nigra on the ipsilesional side. Scale bars represent 50 μm.

Furthermore, we carried out an immunohistochemical stain specific for aggregated α-synuclein. Under high magnification, aggregates were visible in some animals suggesting that FN075 in combination with AAV-α-synuclein does induce α-synuclein aggregation on the ipsilesional side in the case of both the wild-type vector (Figure 5a) and the A53T mutant vector (Figure 5b). 

### 3.3. FN075 Did Not Precipitate Neurodegeneration in the Nigrostriatal Pathway in Preclinical AAV Models

The degeneration of the dopaminergic neurons of the nigrostriatal pathway is a central component of Parkinson’s disease neuropathology. Thus in our post mortem analyses, we sought to determine if administration of FN075 would precipitate neurodegeneration in rats with pathological α-synuclein expression.

**Figure 6:**
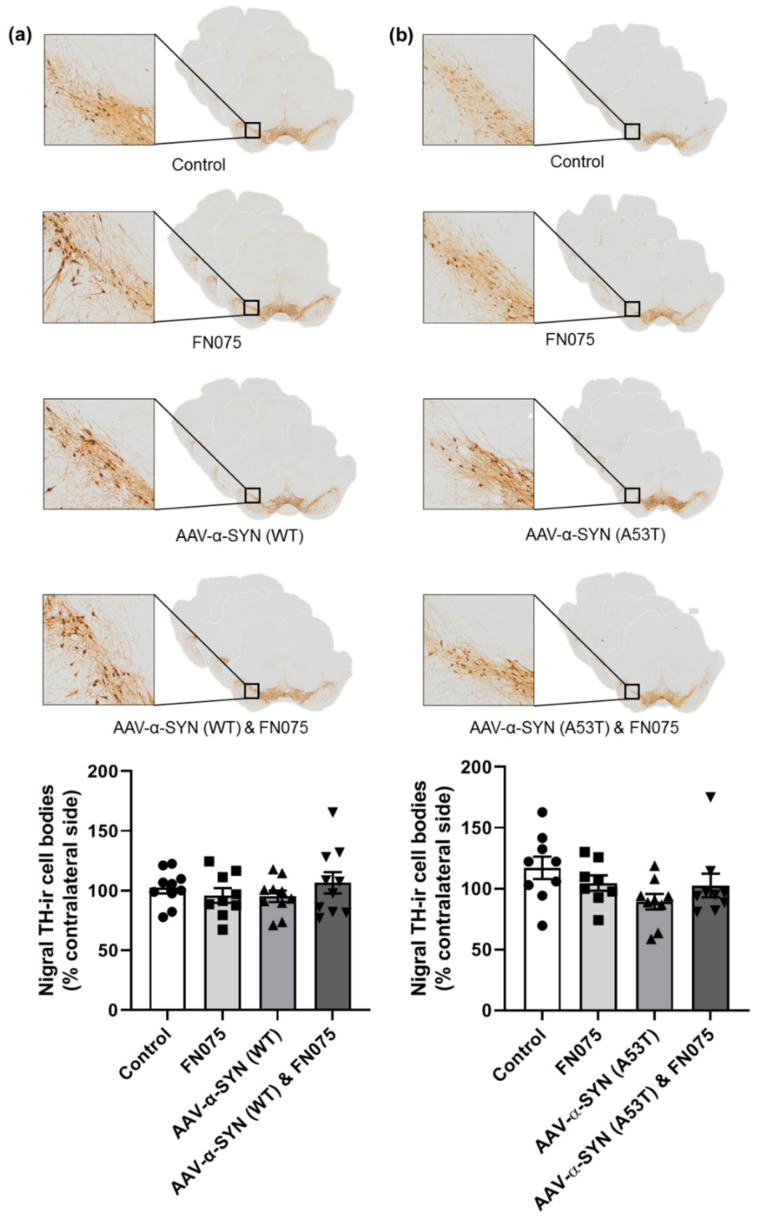
Immunohistological assessment of the impact of AAV-α-synuclein vectors alone or in combination with FN075 on nigral cell counts. (**a**) AAV-α-SYN (WT) or (**b**) AAV-α-SYN (A53T), either alone or in combination with FN075, did not significantly affect the number of TH-immunoreactive cell bodies in the substantia nigra. Data are represented as mean ± SEM with *n* = 8–10 animals per group by one-way ANOVA.

**Figure 7:**
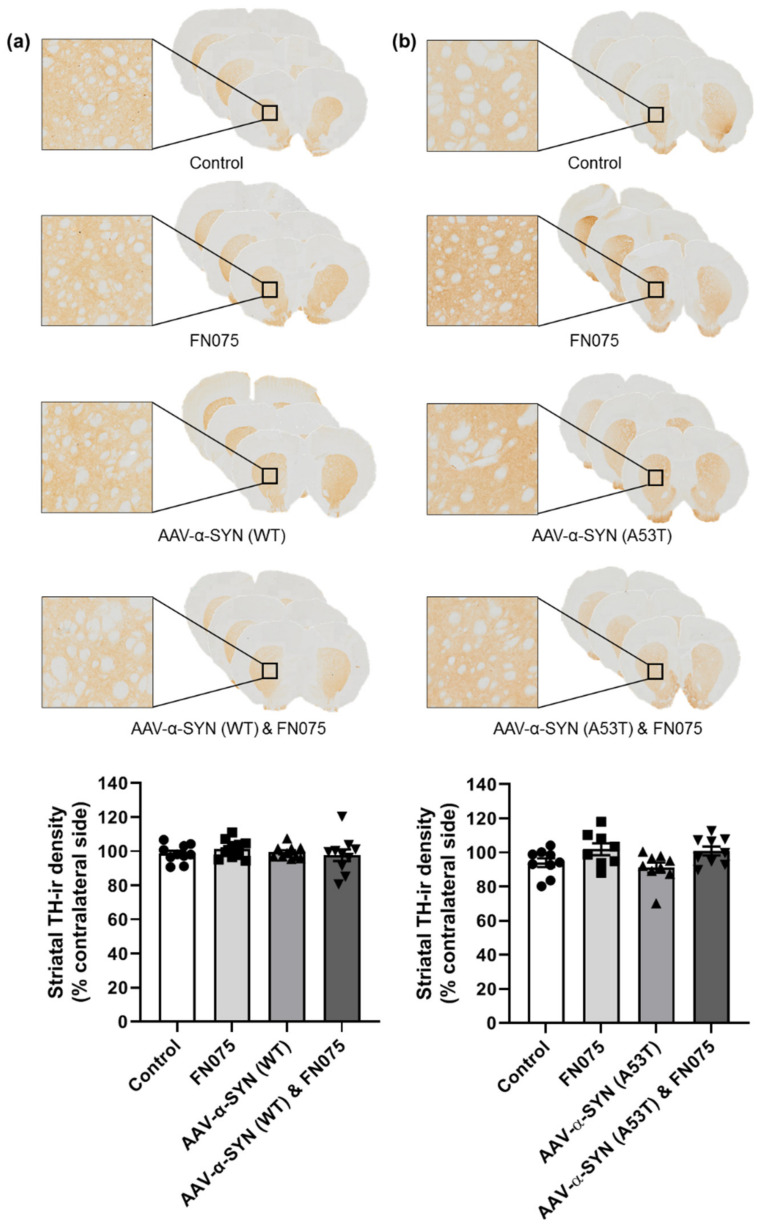
Immunohistological assessment of the impact of AAV-α-synuclein vectors alone or in combination with FN075 on the density of dopaminergic nerve terminals in the striatum. **(a)** AAV-α-SYN (WT) or **(b)** AAV-α-SYN (A53T), either alone or with FN075, did not significantly affect the optical density of TH-immunoreactive staining in the striatum. Data are represented as mean ± SEM with *n* = 8-10 animals per group by one-way ANOVA.

In the substantia nigra, despite the pronounced increase in pathological pS129-α-synuclein accumulation in the combined group, this did not manifest or precipitate an overt loss of nigrostriatal dopaminergic neurons. Thus, in both the wild-type and mutant studies, there was no significant difference in tyrosine hydroxylase immunopositive cell counts in the substantia nigra (WT: Figure 6a; Group, *F*_(*3*,*35*)_ = 0.72, *p* > 0.05; A53T: Figure 6b; Group, *F*_(*3*,*31*)_ = 2.04, *p* > 0.05). Similarly, the single sequential dose of FN075 did not precipitate any loss of tyrosine hydroxylase immunopositive terminals from the striatum in either AAV model (WT: Figure 7a; Group, *F*_(*3*,*36*)_ = 0.50, *p* > 0.05; A53T: Figure 7b; Group, *F*_(*3*,*31*)_ = 3.26, *p* > 0.05). 

### 3.4. FN075 Did Not Precipitate Motor Impairment in the Nigrostriatal Pathway in Preclinical AAV Models

A key feature of a unilateral Parkinson’s disease model is the presence of motor impairment on the side of the body contralateral to the lesion site. The ipsilateral side of the body should not exhibit motor impairment and therefore can serve as an internal control. Thus, in these studies, we assessed the rats in a battery of lateralized motor tests to determine if FN075 could precipitate contralateral motor impairment in the AAV models. 

**Figure 8:**
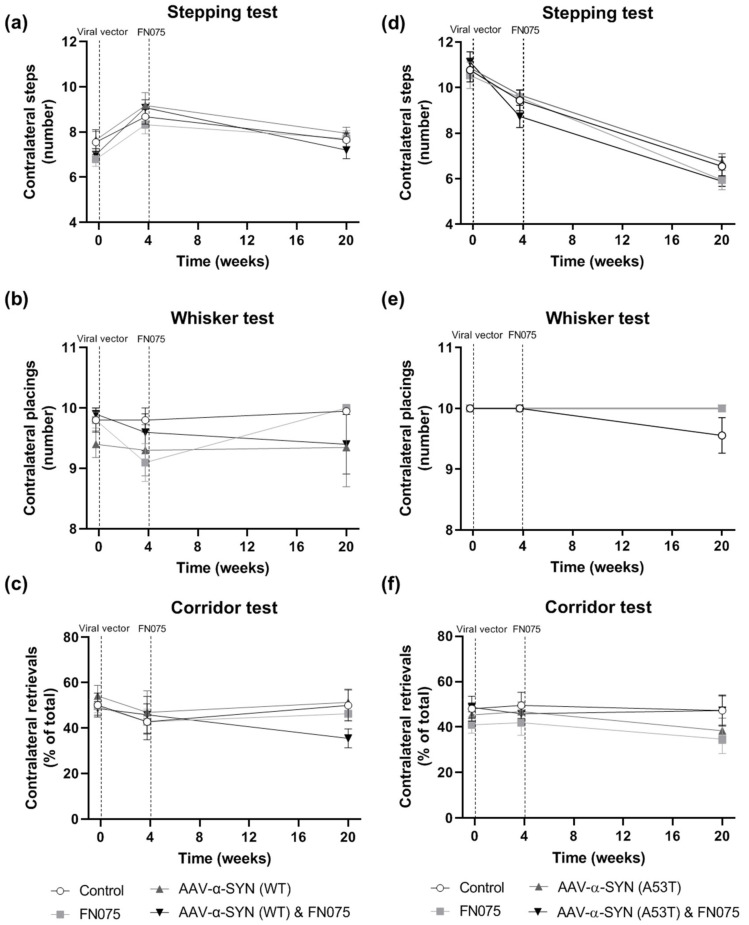
Impact of AAV-α-synuclein vectors alone or in combination with FN075 on contralateral motor function. (**a**–**c**) AAV-α-SYN (WT) or (**d**–**f**) AAV-α-SYN (A53T), alone or in combination with FN075, did not induce contralateral motor dysfunction in the Stepping, Whisker or Corridor behavioral tests. Data are represented as mean ± SEM with *n* = 8–10 animals per group by two-way repeated-measures ANOVA. Dashed lines represent the days of infusion surgeries.

In the wild-type experiment, no significant differences were observed at any time-point in contralateral performance in the Stepping Test of forelimb akinesia (Figure 8a; Group x Time, *F*_(*6*,*72*)_ = 1.09, *p* > 0.05) or the Whisker Test of sensorimotor integration (Figure 8b; Group x Time, *F*_(*6*,*72*)_ = 0.83, *p* > 0.05). There was a slight trend for motor impairment in the Corridor Test of sensorimotor neglect in the AAV-α-SYN (WT) & FN075 group compared to the other 3 groups, but this was not statistically significant (Figure 8c; Group x Time, *F*_(*6*,*72*)_ = 1.07, *p* > 0.05). In the A53T α-synuclein experiment, there were no differences between groups in the Stepping (Figure 8d; Group x Time, *F*_(*6*,*62*)_ = 1.07, *p* > 0.05), Whisker (Figure 8e; Group x Time, *F*_(*6*,*62*)_ = 2.19, *p* > 0.05), or Corridor (Figure 8f; Group x Time, *F*_(*6*,*62*)_ = 0.44, *p* > 0.05) tests. 

## 4. Discussion

Most of the currently available animal models of Parkinson’s disease involve the administration of a toxin, either systemically or directly into the brain, which causes degeneration of dopaminergic neurons in the nigrostriatal pathway. While these models are undeniably useful in the study of some aspects of the disease, they are not entirely representative with regard to the rapidity at which they cause neuronal death and the mechanism by which this death is caused. The establishment of the AAV-α-synuclein model was a major advancement to the field, as it causes α-synuclein overexpression and a gradual neuronal loss [3] that is more representative of the changes occurring in the brains of Parkinson’s disease patients. However, this model is still limited as it takes months to progress, and it is also associated with a very high degree of variability. Therefore, the development of novel animal models of this disease is vital for the future study of the discipline. 

Several attempts have been made thus far to improve upon the AAV-α-synuclein Parkinson’s disease model. The laboratory of Anders Björklund recently obtained promising results for a potential novel model by the combination of preformed fibrils and AAV-α-synuclein, reproducing Lewy body pathology and progressive dopamine cell loss [29]. This synucleinopathy induced a profound inflammatory response, with activation of microglia and infiltration of lymphocytes. Furthermore, a study carried out by a previous member of our research group found that the sequential intra-nigral administration of AAV-α-synuclein and the pesticide rotenone significantly exacerbated nigrostriatal degeneration and induced a progressive decline in motor function [30]. Sequential administration of subclinical doses of intra-nigral AAV-α-synuclein and intra-striatal rotenone similarly precipitated motor impairment and degeneration of nigral cell bodies [31]. Undoubtedly, there is enormous potential in using a dual approach to modelling Parkinson’s disease pathology. 

FN075 is a peptidomimetic small molecule that has been shown to accelerate α-synuclein aggregation, and the oligomers formed by this process are structurally similar to those formed by naturally occurring aggregation [12,13]. Previous studies suggest that FN075, with its peptide-like backbone, promotes α-synuclein templating and oligomerization via an oligomer stacking model [13,32]. In vivo, FN075 caused impairments in motor function and nigrostriatal degeneration at six months in mice after a single injection of the molecule [14]. In rats, sequential exposure to the viral mimetic, poly I:C, followed by FN075 caused an exacerbatory effect on neurodegeneration, neuroinflammation and motor impairment [15].

Given the aforementioned problems with the AAV-α-synuclein model and the results from previous FN075 studies, we decided to attempt to develop a more relevant and more rapidly progressing model of Parkinson’s disease by combining AAV-α-synuclein with FN075, the results of which are presented in this paper. Across two studies, we administered a viral vector that expressed either human wild-type α-synuclein or human α-synuclein with the A53T mutation, and then four weeks later administered the α-synuclein aggregating small molecule FN075. We assessed lateralized motor performance throughout the studies and assessed α-synuclein expression and dopaminergic degeneration post mortem. In both experiments, we observed that the α-synuclein viral vector induced a significant overexpression of human α-synuclein in the nigrostriatal pathway. Furthermore, in both studies, FN075 in combination with the α-synuclein viral vectors caused a significant increase in phosphorylation of α-synuclein, as measured by both optical density analyses and by counts of cells positive for pS129-α-synuclein. However, this α-synuclein overexpression and pathology was not sufficient to induce major nigrostriatal degeneration, which explains the lack of noticeable impairments in the lateralized motor tests. 

As previously stated, the degeneration of the dopaminergic neurons in the nigrostriatal pathway is the main pathological characteristic of the Parkinsonian brain, and the impairments in motor function that are the primary clinical manifestations in Parkinson’s disease patients are a consequence of the loss of these neurons. In these studies, we used a subclinical model of AAV-α-synuclein that did not induce dopaminergic degeneration independently. In these experiments, the sequential administration of FN075 did not precipitate neuronal death in the rats that overexpressed α-synuclein. This highlights the issues that are associated with the AAV-α-synuclein models, as even when they induce widespread α-synuclein pathology, this is not always sufficient to induce consistent degeneration of dopamine neurons [3,11]. An α-synuclein viral vector at a titer that induces dopaminergic neuronal death may be required in order to fully ascertain the potentiality of a combined model with FN075. This approach would allow us to assess if there was an additive effect of FN075 on the extent or rate of neuronal death and motor impairment compared to the standard preclinical AAV-α-synuclein model. 

FN075 is a small molecule that has previously been shown to cause aggregation of α-synuclein [14,15]. In the two studies presented in this paper, FN075 in combination with the AAV-α-synuclein vectors significantly increased phosphorylated α-synuclein compared to the control, as measured by both the optical density analyses and the pS129-α-synuclein accumulation counts. The phosphorylation of α-synuclein is a characteristic trait of Lewy bodies, with 90% of α-synuclein deposited in Lewy bodies extensively phosphorylated at serine 129. In comparison, only 4% of α-synuclein in healthy brains is phosphorylated at this residue [33,34]. Antibodies against ps129-α-synuclein are frequently used as markers of α-synuclein aggregates in the brain, and indeed pS129-α-synuclein is being investigated as a potential biomarker for Parkinson’s disease [35,36,37]. Therefore, it is promising that our novel combined model is consistent with regard to this pathophysiological feature. 

In summary, the combination of AAV-α-synuclein and FN075 holds significant promise as a novel modelling approach, causing enhanced α-synuclein pathology. However, further studies with alternative administration regimes are required to determine the true potential of this model at improving the reliability and variability of these viral-induced α-synuclein overexpression models. 

## Figures and Tables

**Table 1:** Groups used in these two studies. In the first experiment, the AAV vector expressed the wild-type human α-synuclein transgene, while in the second experiment, the AAV vector expressed the A53T mutant human α-synuclein transgene. AAV vectors were injected unilaterally into the substantia nigra followed four weeks later by administration of FN075 at the same site. See text for more methodological detail.

Study 1: Wild-Type Experiment			
**Group**	**Virus injection**	**FN075 injection**	***n***
Control	AAV-GFP	Vehicle	10
FN075	AAV-GFP	FN075	10
AAV-α-SYN (WT)	AAV-α-synuclein (wild-type)	Vehicle	10
AAV-α-SYN (WT) & FN075	AAV-α-synuclein (wild-type)	FN075	10
Study 2: A53T Mutant Experiment			
**Group**	**Virus injection**	**FN075 injection**	***n***
Control	AAV-GFP	Vehicle	9
FN075	AAV-GFP	FN075	8
AAV-α-SYN (A53T)	AAV-α-synuclein (A53T mutant)	Vehicle	9
AAV-α-SYN (A53T) & FN075	AAV-α-synuclein (A53T mutant)	FN075	10

## Data Availability

Research data used in this article are available from the corresponding author on request.
